# Association of Pioglitazone With Major Adverse Cardiovascular Events, All-Cause Mortality, and Heart Failure Hospitalizations: A Systematic Review

**DOI:** 10.7759/cureus.46911

**Published:** 2023-10-12

**Authors:** Ibrahimkhalil M Sheikh, Omar A Hassan, Siad Mohammed Adam, Abdirazak I Ali, Oboseh J Ogedegbe, Godfrey Tabowei, Ahmed Barbarawi, Feisal M Yussuf, Mohammed A Nor

**Affiliations:** 1 General Practice, Erciyes University, Kayseri, TUR; 2 General Practice, Ondokuz Mayis University, Samsun, TUR; 3 General Practice, Baskent University Faculty of Medicine, Ankara, TUR; 4 Pediatrics, University of Minnesota, Minneapolis, USA; 5 Internal Medicine, Lifeway Medical Centre, Abuja, NGA; 6 Internal Medicine, California Institute of Behavioral Neurosciences & Psychology, Fairfield, USA; 7 Internal Medicine, Hennepin County Medical Center (HCMC), Minneapolis, USA; 8 General Practice, Antaliya Hospital, Garissa, KEN; 9 Internal Medicine, Stamford Hospital/Columbia University Vagelos College of Physicians & Surgeons, Stamford City, USA

**Keywords:** heart failure hospitalizations, all-cause mortality, major adverse cardiovascular events, type 2 diabetes mellitus, pioglitazone

## Abstract

Modern diabetic treatment has gone beyond glycemic control, with the choice of different medications to attain therapeutic targets also affected by the risk of long-term outcomes and safety profiles. The effect of diabetes on increased morbidity and mortality and its relationship to cardiovascular outcomes and coronary artery diseases have driven recent diabetes studies toward medications that improve cardiovascular outcomes and reduce all-cause mortality. This is attained by holistically treating cardiovascular complications in type 2 diabetic patients beyond glycemic control. Moreover, both diabetes and pre-diabetes are considered risk factors for both microvascular and macrovascular cardiac events. Despite the fact that initial research acknowledged fluid retention as a safety issue in pioglitazone use, clinical trial data have not presented conclusive proof of a positive or negative impact on cardiac function. This comprehensive literature review aims to evaluate the effect of pioglitazone on all-cause mortality, hospitalizations for heart failure, and major adverse cardiovascular outcomes, including the individual outcomes of non-fatal stroke, non-fatal myocardial infarction, and cardiovascular mortality.

## Introduction and background

The transition from normal glucose tolerance to diminished glucose tolerance and, finally, type 2 diabetes mellitus (T2DM) with the addition of beta cell failure has been thoroughly described in the natural history of T2DM. Cardiovascular disease (CVD) remains the most frequent cause of morbidity and mortality among patients with T2DM. Pioglitazone is an extensively used drug for the treatment of T2DM. It acts predominantly as insulin sensitized in peripheral tissues by binding and activating the nuclear peroxisome proliferator-activated receptor gamma (PPARY) expressed in those tissues [1]. Pioglitazone is also known to have additional favorable metabolic benefits in T2DM patients. In addition to lowering blood pressure, it also enhances lipid profiles in insulin-resistant individuals [2]. Studies have consistently discussed an increased risk of hospitalization for heart failure with pioglitazone due to its well-documented side effect of fluid retention [1]. It has thus remained a popular choice in many parts of the world, especially with its well-publicized insulin sensitization properties [3,4]. Furthermore, counterarguments state that despite its sodium-water retention, it does not lead to a concomitant increase in mortality secondary to heart failure, and demonstrates no adverse effect on the heart [5]. The mechanisms by which these actions are carried out may be dependent on its anti-remodeling properties (inflammation-modulation), metabolic (adipose tissue metabolism, increased high-density lipoprotein (HDL) cholesterol), and neurohormonal (renin-angiotensin-aldosterone system and adiponectin) [1]. This review article extensively discusses the correlation and association between pioglitazone and major adverse cardiovascular events, all-cause mortality, and heart failure hospitalizations.

## Review

Methodology

This systematic review is based on the Preferred Reporting Items for Systematic Reviews and Meta-analyses (PRISMA) 2020 guidelines [6].

Database and Search Strategy

Relevant studies were chosen to evaluate a clear relationship between pioglitazone and significant adverse cardiovascular effects, all-cause mortality, and heart failure hospitalizations. We selected studies from PubMed, Google Scholar, and Cochrane Library, published from 2003 to 2023, including systematic reviews, traditional reviews, reviews of literature, randomized clinical trials (RCTs), and observational studies. Articles published outside the chosen timeframe, unrelated to the topic, not published in English, and abstracts without access to full-text articles were excluded. We used the keywords "Pioglitazone," " major adverse cardiovascular events," " heart failure hospitalizations," and "all-cause mortality". These keywords were combined in all possible combinations to generate articles for screening.

Selection Strategy, Data Collection, and Outcome Assessment

Using the same search technique across both databases, two reviewers seperately reviewed and selected the articles. Articles were initially screened based on their titles and abstracts, and then afterwards by reading the complete text of the papers. When opposing conclusions on the eligibility of an article were found, reviewers evaluated the full-text article until they came to an agreement. The obtained data was scrutinized and then tabulated under the headings of first author, year of publication, type of study, method, limitations, and conclusion.

Analysis of Study Quality

A total of nine articles were chosen for this systematic review. Three of these were systematic review and meta-analyses, four were randomized clinical trials, and two were observational studies. The quality assessment tools used to conduct this systematic review included the PRISMA 2020 checklist [6], the Cochrane Collaboration risk-of-bias tool (CCRBT) [7] for RCTs, and the Newcastle-Ottawa scale for observational studies [8].

Risk of Bias Assessment 

The selected studies were all independently examined and evaluated for risk of bias by two reviewers. This process was executed using the common quality assessment tools for RCTs, systematic reviews, and observational studies. We only admitted studies that scored higher than 70%. The quality assessment tools used for the approved studies are displayed in Table 1.

**Table 1:** Risk of bias assessment

Quality Assessment Tool	Type of Study	Total Score	Accepted score (>70%)	Accepted Studies
Preferred reporting items for systematic reviews and meta-analyses	Systematic Review and Meta-analyses	44	31	Liao et al. [9], Lincoff et al. [10], Zhou et al. [11]
Cochrane collaboration risk-of-bias tool (CCRBT)	Randomized Clinical Trial	7	5	Tanaka et al. [12], Dormandy et al. [13], Mazzone et al. [14], Giles et al. [15]
Newcastle-Ottawa Scale	Observational Studies	8	6	Yang et al. [16], Habib et al. [17]

Results

**Figure 1:**
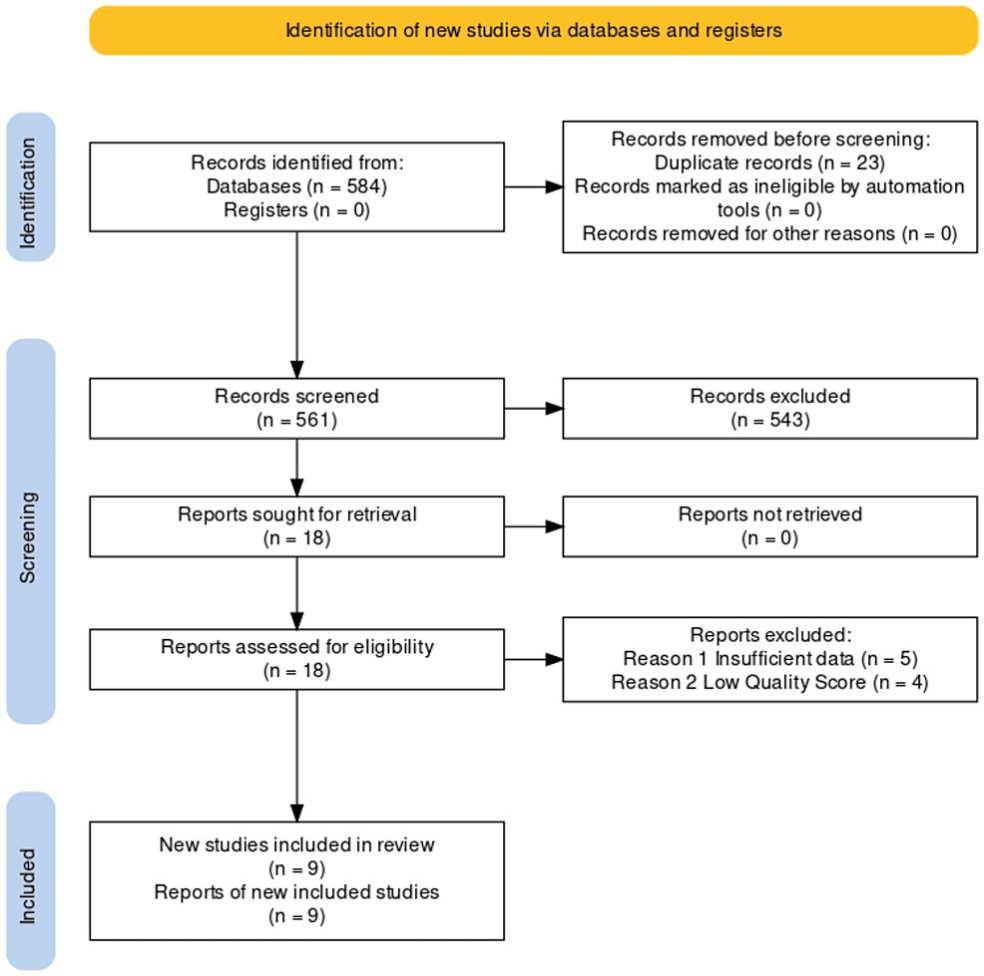
PRISMA flowchart of the study selection process PRISMA: Preferred Reporting Items for Systematic Reviews and Meta-analyses

The initial database search yielded 584 articles with possibly related articles to our topics of interest. We eliminated duplicates and these left 561 articles. Appraisal and assessment of these titles and abstracts of these articles, based on the stated criteria led to the elimination of 543, leaving 18 articles. Nine of these were further removed due to insufficient data or low quality score. The final nine papers had scores >70% when quality assessment was done. These included four RCTs, three systematic reviews and meta-analyses, and two observational studies. Figure 1 describes the study selection and screening process. The characteristics fo the studies are described in Table 2.

**Table 2:** Characteristics of studies included in the review CIMT: carotid intima-media thickness; CVA: cerebrovascular accident; TIA: transient ischemic attack; RR: risk ratio; WMD: weighted mean difference; CI: confidence interval; HR: heart failure; ER: emergency room; CHD: coronary heart disease; CHF: congestive heart failure; TZDs: thiazolidinediones, AMI: acute myocardial infarction; T2DM: type 2 diabetes mellitus

Author	Year of Study	Type of Study	Methods	Limitations	Conclusion
Liao et al. [9].	2016	Meta-analysis	Randomized controlled trials comparing pioglitazone with any control were identified, which included placebo or other glucose-lowering agents. Follow-up was for a minimum of 1oneyear.	Two major randomized controlled trials dominated the findings.	People with insulin resistance, pre-diabetes, and diabetes mellitus (DM) who took pioglitazone experienced reduced major adverse cardiovascular events. The risks of heart failure, edema, and weight gain were, however, raised by pioglitazone. In persons with insulin resistance and pre-diabetes, pioglitazone decreased new-onset DM.
Tanaka et al. [12].	2015	Randomized clinical trial	Participants were divided into two groups: patients who had an ischemic CVA or TIA with or without a history of T2DM, with a diagnosis by 75-g oral glucose tolerance test.	The power of this investigation was insufficient to determine pioglitazone effects. This is because the study population was just 120.	According to this study, patients with newly diagnosed diabetes and reduced glucose tolerance who had experienced an ischemic stroke or transient ischemic attack did not see any positive outcomes with pioglitazone use.
Dormandy et al. [13].	2005	Randomized Clinical Trial	Participants were given oral pioglitazone 15 mg to 45 mg and other oral hypoglycemics in a randomized controlled study involving 5238 participants. All-cause mortality, nonfatal myocardial infarction, and stroke comprised the primary outcome.		In patients with T2DM with a high risk of macrovascular events, pioglitazone lowers the risk of all-cause mortality, non-fatal myocardial infarction, and stroke.
Lincoff et al. [10].	2007	Meta-analysis	A database of patient clinical data from 19 clinical studies and a patient population of 16390 was examined to assess the effectiveness and safety of pioglitazone when administered alone, with insulin or in combination with other hypoglycemic medications. The primary outcomes of this investigation were nonfatal myocardial infarction, nonfatal stroke, and death from any cause.	Endpoints were not consistently determined or measured using conventional criteria since most of the trials combined for this analysis were not initially designed to evaluate cardiovascular outcomes. In addition, some trials were excluded because databases and data were lacking.	Among a wide range of diabetes patients, pioglitazone is associated with a noticeably lower risk of death, myocardial infarction, or stroke. Pioglitazone increases severe heart failure, although there is no corresponding rise in mortality.
Mazzone et al. [14].	2006	Randomized Clinical Trial	A randomized, double-blind, comparator-controlled, multicenter trial for people with T2DM using pioglitazone hydrochloride (15-45 mg/d) or glimepiride (1-4 mg/d) as an active comparator was conducted at 28 clinical sites.	Because the trial did not have enough power to detect a difference in cardiovascular endpoints, it could not prove that treatment with pioglitazone rather than glimepiride will lower these endpoints in people with T2DM. Additionally, the study had a dropout rate of about 30%.	Over an 18-month medication period, pioglitazone in patients with T2DM retarded the evolution of CIMT in contrast to glimepiride.
Zhou et al. [11].	2020	Meta-Analysis	The study discovered RCTs contrasting pioglitazone with any control. Cardiovascular and renal outcomes were gathered, including changes in the urinary albumin to creatinine ratio and protein excretion throughout a 24-hour period. Additionally pooled were the RR and WMD, both with 95% CIs.	The eligible RCTs varied regarding participant baseline characteristics, sample sizes, or combined treatments, which caused heterogeneity. A small number of the trials that were pooled for this analysis were not initially intended to assess cardiovascular or renal outcomes; as a result, endpoints weren't arbitrated consistently or evaluated according to standard standards.	To prevent cardiovascular outcomes, pioglitazone should be explored in patients with or at high risk of T2DM, particularly those with a history of existing CVA who would benefit most.
Yang et al. [16]	2014	Observational Study	Using Cox proportional hazards models adjusted with inverse probability weights derived from propensity scores, Kaplan-Meier curves were produced, and hazard ratios (HRs) were calculated for the occurrence of deaths in the pioglitazone and insulin cohorts.	There was a lack of reliable data on the variations in disease worsening rates and several clinical and laboratory findings, which restricted the statistical adjustment of baseline variables.	Compared to insulin, pioglitazone was linked to a decreased risk of overall mortality.
Habib et al. [17].	2009	Observational Study	A retrospective cohort research was conducted between January 1, 2000, and December 31, 2006. AMI, both fatal and non-fatal, was the main result. CHF hospitalizations, fatal and non-fatal CVA, TIA, combined CHD occurrences, and all-cause death were considered secondary outcomes.	The observational nature of the study may have resulted in significant treatment group differences that were not taken into account by the regression models.	Results argue against a single effect of TZDs on cardiovascular outcomes, with pioglitazone perhaps having a better risk profile when compared to rosiglitazone.
Giles et al. [15].	2009	Randomized Clinical Trial	In this double-blind, randomized, multicenter research, participants received pioglitazone or glyburide (insulin) for six months. Time to HF, a composite of cardiovascular mortality and hospitalization or ER visit for HF, was the main outcome. Echocardiographic and functional categorization tests were secondary objectives.	The trial included a two-week screening phase during which oral hypoglycemics were not administered, and a quick dose titration plan that deviated from the pioglitazone dosage guidelines for clinical usage.	Pioglitazone was linked to a greater incidence of HF hospitalizations without deteriorating echocardiographic heart function or cardiovascular mortality.

Discussion

Pioglitazone and Major Adverse Cardiovascular Events (MACE)

Studies have consistently shown that insulin resistance and T2DM increase the risk of myocardial infarction (MI) and stroke. This is due to a link between the pathophysiology of diabetes, inflammation, and high blood pressure, which all increase the chances of developing CVDs [9]. The United States Food and Drug Administration (FDA) and the European Medicines Agency (EMA) both published guidelines on using the three-point MACE outcome, which includes acute MI, stroke, and cardiovascular mortality in all trials appraising the cardiovascular safety of diabetic agents, in 2008 and 2012, respectively [18] In addition, some studies have adopted a four-point MACE, including hospitalization for unstable angina or revascularization procedures, and a five-point MACE, including heart failure [19]. 

Liao et al. conducted a systematic review and meta-analysis among patients with insulin resistance, pre-diabetes, and T2DM. The risks of MACE and MI were found to be decreased for pioglitazone in this study (risk ratio (RR) 0.77, 95%CI 0.64 to 0.93; p-value for heterogeneity=0.44, I2=0%) [9]. Pioglitazone was associated with a propensity to reduce the incidence of recurrent stroke in patients with pre-diabetes or insulin resistance (RR 0.81, 95% CI 0.65 to 1.01; p for heterogeneity=0.45, I2=0%). Kaku et al. supported this fact in a prospective study which concluded that pioglitazone caused a delay in the time of onset of macrovascular events and was also linked with a lower cumulative incidence for macrovascular events (3.56% vs. 4.49% for controls) [20]. The J-spirit study concentrated on just one of the MACE outcomes and decided that pioglitazone was associated with lower outcomes for the primary endpoint of stroke recurrence than in the control group (hazard ratio (HR) =0.62, 95%CI 0.13-2.35, p=0.49) [12]. Even though the exact etiological factors responsible for the beneficial effect of pioglitazone towards cardiovascular risk reduction are unknown, this is unlikely to be due to its hypoglycemic effect [21]. The etiopathogenesis of atherosclerosis is multi-factorial, resulting from dyslipidemia, lipoprotein oxidation, and inflammatory cellular actions [22]. In all likelihood, pioglitazone possesses a pleiotropic effect on cardiovascular risk reduction.

Several extensive studies have shown the effect of pioglitazone on cardiovascular outcomes. In PROactive (PROspective pioglitazone Clinical Trial In macroVascular Events), a multicenter, randomized study, patients were treated using guidelines-specific therapy for a variety of risk factors in order to ascertain the effects of pioglitazone on macrovascular outcomes. In comparison to placebo, pioglitazone was linked with a favorable trend for macrovascular endpoints (19.7% vs. 21.7%; HR = 0.90, 95%CI 0.80-1.02, P = 0.095) [13]. Furthermore, a remarkable outcome with MACE composite endpoints was also seen, specifically the pre-determined combination of cardiovascular mortality, MI, and stroke (9.9% vs. 11.9%; HR = 0.82, 95%CI 0.70-0.97, P = 0.020) [23]. A considerable decrease in the risk of recurrent MI was also seen (5.3% vs. 7.2%; HR = 0.72, 95%CI 0.520.99, P = 0.0453) and recurrent stroke (5.6% vs. 10.2%; HR = 0.53, 95%CI 0.34-0.85, P = 0.009) [24]. This shows that pioglitazone may have the ability to stabilize plaque.

Similar to this, a meta-analysis of RCTs examining 19 trials with 16,390 patients revealed that MACE occurred in 450 of 7836 patients receiving control therapy and 375 of 8554 patients receiving pioglitazone (4.4%) and 5.7%, respectively (HR = 0.82; 95%CI 0.72-0.94; P = 0.005) [10]. The most prominent clinical study focusing on pioglitazone's effect on intima-media thickness is the CHICAGO (Carotid Intima-media Thickness in Atherosclerosis using pioglitazone) study. This prospective randomized multi-center compared the effect of pioglitazone in beneficially decreasing carotid intima-media thickness (CIMT) vs. glimepiride. Pioglitazone showed a 14% increase in high-density lipoprotein (HDL) cholesterol and subsequently showed a significant decline in the progression of CIMT compared to glimepiride [14]. Several large, extensively designed clinical trials have provided recent significant data on the consequences of pioglitazone on MACE. The Insulin Resistance Intervention after Stroke (IRIS) trial, a multicenter, double-blind trial, scrutinized pioglitazone's impact on subsequent cardiovascular events in individuals with insulin resistance who had recently suffered from an ischemic stroke or transient ischemic attack [25]. Studies have shown that pioglitazone may not have an equal effect on all aspects of MACE. In a meta-analysis by Zhou et al., however, in this same trial, pioglitazone was seen to cause a significant reduction in three-point MACE with an OR of 0.85 (95%CI 0.74-0.97) and a p-value of 0.020 [26]. Unlike some other studies, Zhou et al. concluded that while pioglitazone provided benefit in the reduction of MACE in patients with a history of established CVD (RR 0.8, 95%CI 0.7-0.9, p<0.001), there was no evidence of benefit in patients with no history of CVD (RR 1.0, 95%Cl 0.7-1.3, p= 0.709 [11]. The risk of non-fatal MI was notably reduced in patients on pioglitazone by 22% (RR 0.8, 95%CI 0.6-1.0, p = 0.023) and non-fatal stroke by 19% (RR 0.8, 95%CI 0.7-1.0, p = 0.018) in patients with a history of established CVDs. However, no significant decrease was seen in those with multiple risk factors, without overt CVD (RR 0.9, 95%CI 0.6-1.5, p = 0.768; RR 0.8, 95%CI 0.4-1.4, p = 0.355) [11].

Nevertheless, how does pioglitazone carry out these actions to reduce MACE? Studies have shown that numerous pathogenic mechanisms that contribute to the emergence of CVD are attacked by pioglitazone. The metabolic syndrome, which is linked to an elevated risk of vascular events and mortality, is characterized by the hallmark feature of insulin resistance, which has a vital role in the development of hypertension, hyperlipidemia, and diabetes [27]. Pioglitazone, a peroxisome proliferator-activated receptor γ (PPARγ) agonist, is said to have an anti-atherosclerotic effect. PPAR-γ is present copiously in adipocytes, which are responsible for modulating adipocyte differentiation. However, it is also present in other cells engaging in vascular injury, such as endothelial cells, vascular smooth muscle cells (VSMCs), and macrophages [28]. Both insulin resistance and systemic low-grade inflammation are etiological factors related to atherosclerotic plaque formation, and pioglitazone ameliorates insulin resistance and decreases systemic inflammation by reducing plasma adipocytokines, inflammatory markers, and procoagulant factors [29]. PPAR regulates monocyte recruitment to endothelial cells [16], which also affects the inflammatory response in monocytes/macrophages and VSMCs and prevents the production of macrophage foam cells and VSMC proliferation and migration [16]. 

Pioglitazone and All-Cause Mortality 

At this point, while the effect of pioglitazone on MACE has been extensively discussed, it is essential also to explore its effect on all-cause mortality. Patients with diabetes risk dying from any cause nearly double that of people without diabetes after accounting for variables including sex, age, and body mass index (BMI) [17]. A retrospective cohort study by Yang et al. showed an adjusted HR for pioglitazone versus insulin to be 0.33 (95% CI 0.31-0.36) [16]. Furthermore, the pioglitazone group had significantly lower all-cause mortality rates than the insulin group in each subgroup that was looked at, including sex, age, baseline congestive heart failure status, baseline lipid-altering medication use, and baseline metformin use [16]. Habib et al. were in agreement with this conclusion in their retrospective cohort study involving 19,171 patients when they concluded that all-cause mortality was found to be lower among those who used pioglitazone (adjusted HR with propensity adjustment (PA) 0.60, 95%CI 0.42-0.96) [17]. In a systematic review and meta-analysis of randomized clinical trials by Liao et al., results showed no notable variation in the rate of all-cause mortality (RR 0.93, 95%CI 0.80-1.09, p for heterogeneity=0.88, I2=0%) [9], while a meta-analysis by Zhou et al. showed no applicable effect on all-cause mortality (RR 1.0, 95%CI 0.8-1.2, P = 0.64; RR 1.1 (95%CI 0.7-1.5), P = 0.78) [11]. A similar conclusion was reached by Mannucci et al., who carried out a meta-analysis on 20 RCTs with 19,779 patients regarding the effect of glucose-lowering agents on cardiovascular outcomes and postulated that pioglitazone was neutral towards all-cause mortality, with an OR of 0.93 (95%CI 0.78-1.11, p-value = 0.39) [26]. Yen et al. added some perspective to this discussion, postulating that in insulin-treated patients, pioglitazone reduces the likelihood of all-cause mortality. In their study, a lower risk of non-cardiovascular death was the key factor contributing to the decreased mortality rate [30]. When comparing pioglitazone users to nonusers, the adjusted HR of mortality was 0.47 (95%CI 0.38-0.58, P = 0.001) [30]. Reasons that have been postulated for this decreased mortality include that the use of pioglitazone may reduce insulin requirement and subsequently, dosage, with lower insulin, leading to a reduced rate of atherosclerotic changes induced by hyperinsulinemia [31], increase in mean low-density lipoprotein (LDL) particle size [32], and reduction in inflammatory factors [33].

Pioglitazone and Heart Failure Hospitalizations

Heart failure and T2DM frequently coexist, significantly affecting clinical care and prognosis. Compared to heart failure patients without T2DM, patients with heart failure with reduced ejection fraction (HFrEF) and preserved ejection fraction (HFpEF) have worse clinical states and higher all-cause and cardiovascular mortality [12]; however, a direct causal relationship is yet to be established and pioglitazone's usage in patients with heart failure is therefore constrained since studies repeatedly demonstrate that it causes fluid retention, frequently interpreted as decreasing cardiac function [34]. Although several surrogate measures of cardiovascular risk have improved with its treatment, usage is constrained by the dose-dependent, typically mild to moderate, fluid retention seen with these drugs. In a randomized clinical trial by Giles et al., pioglitazone was compared to glyburide for the primary outcome of time to heart failure, a composite of cardiovascular mortality and hospitalization or emergency room visits for heart failure. Echocardiographic and functional categorization tests were secondary outcomes [15]. Pioglitazone (13%) was shown to have a faster time to onset and a greater incidence of the primary outcome than glyburide (8%) (P =.024) [15]. Karter et al., in a cohort study, add a different perspective based on timeframe to this discussion by elucidating that when short-term pioglitazone use was compared to the conventional, first-line diabetes treatment such as sulphonylureas, metformin, and insulin, there was no evidence of a higher risk of congestive heart failure hospitalization (HR = 1.28; 95%CI 0.85-1.92) [35]. When compared to the control group, pioglitazone was found to be associated with a greater risk of heart failure in a systematic review and meta-analysis by Liao et al. (RR 1.32, 95%CI 1.14-1.54; p-value for heterogeneity = 0.43, I2=0%) [9].

In a meta-analysis of RCTs seeking to evaluate the effect of pioglitazone on ischemic cardiovascular events, 200 (2.3%) pioglitazone-treated patients and 139 (1.8%) control patients experienced severe heart failure (HR = 1.41, 95%CI 1.14-1.76, P = 0.002) [10]. Zhou et al. conducted a meta-analysis on RCTs. They concluded that pioglitazone, in contrast with the control, increased the risk of hospitalization due to heart failure by 33% (RR 1.3, 95%CI 1.1-1.6, p-value < 0.01), among patients with a background of known CVD [16]. However, in patients without established CVD, no statistically significant association was seen, although these patients had numerous risk factors (RR 1.5, 95%CI 0.8-2.9, p-value = 0.22) [10]. This shows that pioglitazone may not increase the risk of heart failure hospitalization in patients without established CVD. This was confirmed by a meta-analysis of RCTs by Mannucci et al., which revealed that pioglitazone was connected to a higher incidence of heart failure hospitalization, with OR of 1.30 (95%CI 1.04-1.62; p-value = 0.020). Similar to this, a retrospective cohort research by Habib et al. found a statistically significant association between congestive heart failure and pioglitazone, with an HR of 1.24 (95%CI 1.07-1.44) [17].

## Conclusions

The consensus is that while pioglitazone reduces the risk of MACE, it has no treatment effect on all-cause mortality while increasing the risk of heart failure hospitalization. Pioglitazone is a good choice for patients with or at increased risk of T2DM to prevent cardiovascular endpoints, particularly with a co-existing CVD history, as this subset of patients is more likely to have the most significant benefit. Such benefits with a reduction in MACE may only be found in patients with established CVD, even if other patients without CVD have risk factors. Also, these benefits in reducing MACE are equally robust in patients with established T2DM, pre-diabetes, and insulin resistance. Pioglitazone generally does not have a statistically significant effect on all-cause mortality. However, this association shows statistical significance in patients with concurrent insulin use. Further studies are required to scrutinize the possible mechanisms behind the reduction of MACE by pioglitazone and, by extension, the inability to reduce all-cause mortality.
